# Direct growth of ZnO nanowires on civil engineering materials: smart materials for supported photodegradation

**DOI:** 10.1038/s41378-019-0102-1

**Published:** 2019-11-18

**Authors:** Marie Le Pivert, Romain Poupart, Martine Capochichi-Gnambodoe, Nathan Martin, Yamin Leprince-Wang

**Affiliations:** Université Paris-Est, ESYCOM (CNRS-FRE2028), UPEM, 5 Boulevard Descartes, 77420 Champs sur Marne, France

**Keywords:** Nanowires, Nanowires

## Abstract

Photocatalysis is one of the most promising processes for treating air and water pollution. Innovative civil engineering materials for environmental depollution by photocatalysis have already been synthesized by incorporating TiO_2_ or ZnO nanoparticles in cement. This method suffers from two flaws: first, most of the NPs are incorporated into the cement and useless for photocatalysis; second, rain and wind could spread the potentially carcinogenic nanoparticles from the cement surface into nature. Thus, we propose the efficient synthesis of nontoxic and biocompatible ZnO nanostructures solely onto the surface of commercially available concrete and tiling pavements by a low-cost and low-temperature hydrothermal method. Our samples exhibited enhanced photocatalytic activity for degrading organic dyes in aqueous media, and dye molecules are commonly used in the pharmaceutical, food, and textile industries. Durability studies showed no loss of efficiency after four photocatalysis experiments. Such supported structures, which are easy to implement onto the varying surfaces of commercially available materials, are promising for integration into civil engineering surfaces for environmental depollution in our daily life.

## Photocatalysis: Synthesizing safe zinc oxide nanowires for civil engineering materials

An easy, low-cost method has been developed for successfully synthesizing zinc oxide (ZnO) nanostructures onto commercially available materials for environmentally safe use in civil engineering. Photocatalysis is the process whereby light accelerates a chemical reaction, and it holds promise as a means of tackling pollution caused by civil engineering materials. Previous efforts to produce such materials by incorporating ZnO and titanium oxide (TiO_2_) nanoparticles for photocatalysis have suffered drawbacks, notably the nanoparticles being incorporated within the cement and thus useless for photocatalysis. A team headed by Yamin Leprince-Wang at Université Paris-Est, France developed a low-cost, low-temperature hydrothermal method for efficiently synthesizing nontoxic, biocompatible ZnO nanowires solely onto the surface of commercially available concrete, which allowed for excellent photocatalytic efficiency. The team believes that its method has good potential for environmentally safe application on road surfaces.

## Graphical abstract

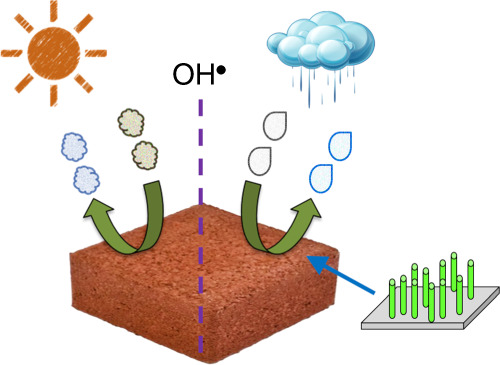

## Introduction

In recent years, water and air pollution caused by industry, urban effluents, and transportation have become a major issue in every urban center. In particular, transportation is responsible for local and extended air pollution, as well as soil and water pollution, affecting biodiversity, human health, and climate. For those reasons, it is important to treat pollution directly at its source, e.g., directly on roads.

To address this issue, the French Institute of Science and Technology for Transport, Development and Networks (IFSTTAR) initiated the development of the 5th generation road (R5G), which intends to be a smart road of the near future^1,2^. The prior four road generations are the mule track (1st generation), Roman roads (2nd), John Loudon McAdam’s tar-coated roads (3rd), and the current roads with improvements enabling haulage and highways (4th)^1^. The R5G will be smartly designed, with new and possibly bio-based materials and the ability to produce energy for urban lighting and/or have sensors to both evaluate pollution levels and detect damaged areas (cracks, e.g.) to replace them before incidents. Last but not least, this new type of road aims to depollute the environment as a “self-cleaning road”. For such a goal, the “I-Street” project was started in 2017^3^, and depollution is one of its top priority axes. To enable our future roads to perform depollution, photocatalytic processes run by UV light and then sunlight are envisaged. Photocatalytic processes are a promising emerging solution for such aim, as they are inexpensive and able to quickly degrade toxic organic compounds into harmless products, such as CO_2_, H_2_O, NO_3_^−^, or SO_4_^2−^. Thus, incorporating chalcogenide and nontoxic semiconductors onto civil engineering materials would be a smart way to achieve this goal, as they are known as excellent photocatalysts^4,5^.

The most widely used semiconductors in photocatalysis are titanium dioxide (TiO_2_) and zinc oxide (ZnO) due to their excellent photocatalytic properties provided by their large bandgap (>3.2 eV) and their band-level potential energy. TiO_2_ has notably received much attention in recent decades. However, this metal oxide has recently become a more controversial material, as it is suspected to be carcinogenic^6,7^. Unlike TiO_2_, ZnO is of particular interest because it is nontoxic and biocompatible^8^. Moreover, ZnO is a remarkable semiconductor, possessing unique properties with a direct wide gap of 3.37 eV at room temperature, a high exciton-binding energy of 60 meV, and low-cost raw materials. Finally, comparison studies have demonstrated that ZnO is a more active photocatalyst than TiO_2_^9–11^. Our research group has developed and optimized an inexpensive and straightforward hydrothermal process to grow well-aligned ZnO nanowires (NWs) onto conventional substrates^12–14^. Such nanostructures are known to have a high surface-to-volume ratio with a high surface defect density, the latter being of crucial importance for the efficient generation of hydroxyl ions in photocatalysis mechanisms.

Even with our efficient synthesis method, incorporating our nanostructures into a road is still an issue. The usual solutions found in the literature are the use of a paint containing the photocatalyst nanoparticles (NPs) or mixing the photocatalyst NPs (TiO_2_ or ZnO) directly into the concrete^15,16^. Those solutions have disadvantages, as incorporated NPs cannot exhibit their optimal photocatalytic efficiency because they are covered by the concrete or paint, and a photocatalytic process requires the semiconductor surface to be in contact with the medium to purify it^17^. To benefit from their optimal efficiency as photocatalysts for organic compound depollution^18,19^, we investigated the direct growth of ZnO nanostructures onto commercially available civil engineering materials. These new construction materials can be used for pavement, which is commonly used in public roads, gardens, and dwellings, and the incorporation of ZnO nanostructures on the surface (only) will enable these structures to photodegrade organic compounds under appropriate light irradiation (artificial and/or sunlight), thus reducing the pollution of the involved medium (air and/or water, namely). In this work, we describe the first reported synthesis of ZnO NWs onto commercially available concrete and tiling pavements as substrates via a low-cost and low-temperature hydrothermal process. Such nanostructures, directly grown onto nonconventional surfaces, were thoroughly characterized by scanning electron microscopy (SEM) and UV–vis spectroscopy to show their microstructures and optical properties and tested in the photocatalytic degradation of three different organic dyes (MO, AR14, and MB) in aqueous solutions.

## Results and discussion

### Characterization

**Fig. 1:**
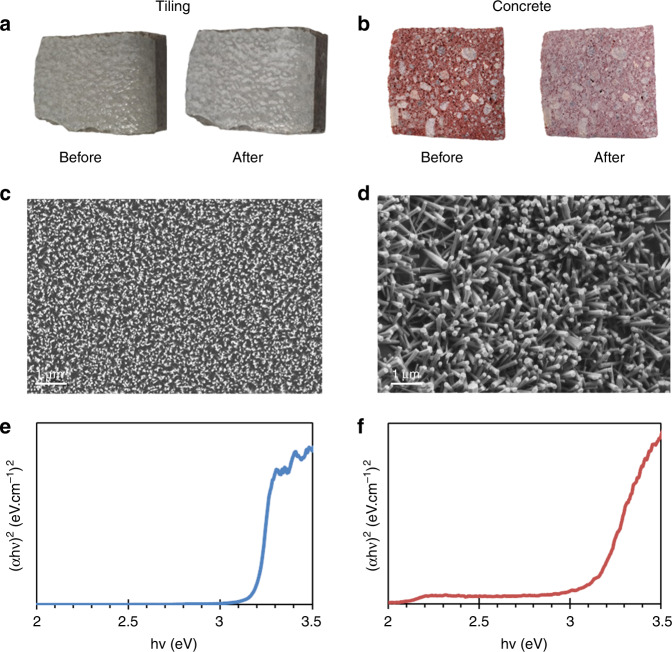
Characterization of ZnO-nanostructure-decorated civil engineering materials. Top views of the gray tiling **a** and red concrete pavement **b** before and after the growth of ZnO nanostructures; SEM top-view pictures showing: **c** ZnO nanowires on the tiling surface, **d** ZnO nanorods and nanostructures on the concrete surface; UV–visible spectral plot with the Tauc–Lorentz model of the sample surfaces: **e** ZnO-decorated tiling and **f** ZnO-decorated concrete

Figure 1a, b shows both samples before and after ZnO NW growth, respectively. We can note that the appearance of the samples was slightly altered; the samples appeared more whitish due to their surfaces being covered by a ZnO NW layer. The microstructure of the as-grown ZnO NWs on those substrates was examined by SEM.

As shown in Fig. 1c, we obtained highly homogeneous NWs on the floor-tiling surface with a diameter of 55 ± 17 nm and a density of ~31 NWs/µm². Considering the substrate mechanical resistance, the classical cleavage method for monocrystalline substrates (such as Si) is not suitable here, and thus, no SEM cross-section view is available here. However, thanks to a model developed by Erfan et al.^20^, the nanowire length on the tiling substrates was estimated by UV–visible measurement and is ~1.794–1.930 μm, depending on the model fitting used. This kind of surface is quite close to the ideal Si–wafer surface, i.e., a smooth and non-porous surface, which explains the almost identical morphology that was observed by SEM^13,17,20,21^. The good homogeneity of the ZnO NWs was also confirmed by the UV–vis measurements, which showed good signal reproducibility all over the substrate. The gap value of our ZnO NWs, calculated according to the Tauc–Lorentz model, is ~3.20 ± 0.03 eV (Fig. 1e). Moreover, this gap value is reproducible anywhere on the substrate and for different tiling samples, demonstrating the ability of hydrothermal ZnO growth to cover commercial materials similar to tiling.

As depicted in Fig. 1d, even though our concrete samples have an extremely rough and porous surface, we obtained a very suitable ZnO NW coverage on their whole surfaces. However, the obtained structures are completely different from the ones grown on a model surface such as Si wafer (i.e., ZnO NWs)^22^ and are larger, such as nanorods with diameters between 100 nm and 400 nm, and a density of 5.2 NWs/µm². Occasionally, other nanostructures could be observed, such as nanoflowers. In addition, due to the chaotic surface, the ZnO nanostructures were oriented in various and random ways. These various heterogeneities in shape, size, and orientation of the ZnO structures could lead to reflection signals, as seen in the UV–vis spectral signals (Fig. 1f) and, thus, to a slightly perturbed signal. However, a gap could still be measured and calculated with the Tauc-Lorentz model, i.e., ~3.18 ± 0.04 eV, which demonstrates the good efficiency of the hydrothermal growth on any of our substrates and the availability of these materials as photocatalysts under UV irradiation.

### Photocatalysis of organic dyes

As described in a previous work^17^, due to their high surface-to-volume ratio, ZnO NWs grown on a Si wafer showed excellent efficiency by decomposing AR14, MB, and MO into less harmful products such as CO_2_, H_2_O, NO_3_^−^, or SO_4_^2−^ via a photocatalytic-induced mineralization process. To measure and prove the depollution efficiency of our new innovative engineering materials, these three organic dyes were used again for our photocatalysis experiments.

**Fig. 2:**
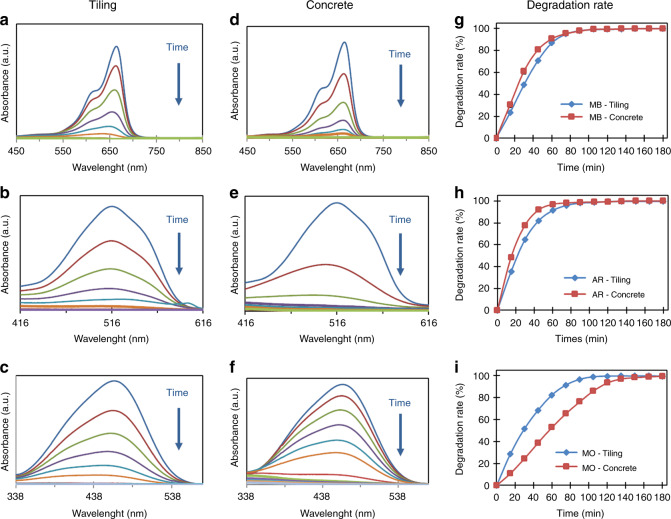
UV–visible investigation of photodegradation of different dyes (MB, AR 14, and MO) using ZnO-NW-decorated tiling and concrete samples as photocatalysts. **a**, **b**, **c** UV–vis spectra recorded during degradation with the tiling substrate for MB, AR 14, and MO, respectively. **d**, **e**, **f** UV–vis spectra recorded during degradation with the concrete substrate for MB, AR, and MO, respectively. **g**, **h**, **i** Different plots of the degradation rate as a function of time for each dye

Figure 2a–f shows the UV–visible spectra recorded during the photodegradation of MB, AR14, and MO with both samples. For every dye, the intensity of the characteristic adsorption peak decreased gradually with increasing UV exposure time, proving that our samples enable the photocatalytic degradation process to take place.

**Fig. 3:**
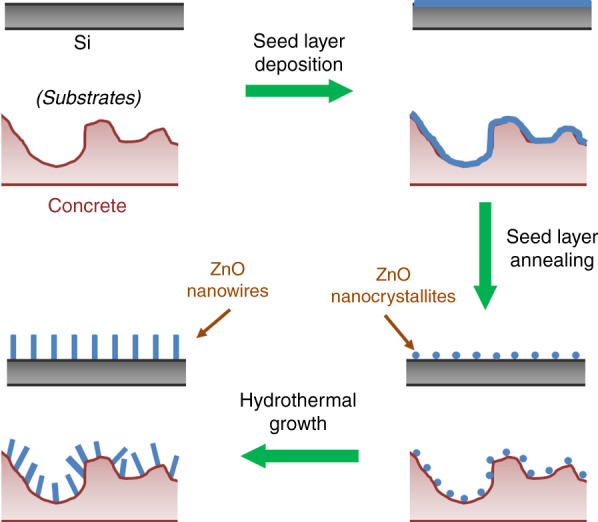
Substrate roughness influence on ZnO NW morphology: scheme of the ZnO NW formation and growth onto a flat substrate (i.e., Si wafer) vs a construction material (i.e., concrete)

The concrete substrates showed a faster dye degradation rate; only ~105 min was needed to achieve total degradation of MB and AR14 (Fig. 2g, h). Similar ZnO NWs on Si wafer samples required ~3 h to reach the same degradation rate^17^. In fact, as shown in Fig. 3, the dendritic structure of the sample increases its specific area, which could lead to better photocatalytic efficiency. This rough surface could also favor dye diffusion by creating micro-turbulence near the ZnO NW surface, improving the pollutant mass transfer and thus the photocatalytic efficiency. In our case, MO, the most difficult to degrade of our model pollutants due to its stability^17,23,24^, was degraded in 120 min with our tiling samples and 150 min with our concrete samples (Fig. 2i), compared with the 3 h needed to obtain an X value of ~95% for the ZnO NWs grown on a Si wafer (Si wafer surface of 2.25 cm² with a ZnO NW length of 1100 ± 50 nm, diameter of 85 ± 5 nm, and density of 23 ± 5 NWs/µm^2^)^25^. The increase in the MO degradation rate due to a combination of ZnO nanorods and a rough and/or porous substrate, such as polyester fiber membranes or a highly porous ceramic substrate (1 cm × 1 cm × 1 cm), was already proven by Danwittayakul et al., with degradation rates of 32 and 77%, respectively, achieved after 3 h under UV-C irradiation^26^. Beyond those previous results, in Fig. 2i, we can note that the tiling substrate leads to faster MO degradation than the concrete substrate. A possible explanation for the reversal of the observed trend is that, in our case, the difficult photocatalytic degradation of MO is more affected by the overall quality of the ZnO nanostructures than by the larger specific area. In fact, it has already been proven that MO is more degraded by the nonpolar surfaces of ZnO than by its polar surfaces^27^.

As shown by the SEM results, the tiling substrate appears to show denser and smaller NWs than other structures, in contrast to the concrete substrate. Therefore, due to a better surface/volume ratio, the ZnO nanostructure-decorated tiling seems to have more nonpolar surfaces corresponding to the six facets on the length than ZnO nanostructure-decorated concrete.

On the other hand, the chemical structure of concrete could also explain the reversal trend; concrete is mostly composed of porphyry and feldspar^28^, which are supposed to favor AR and MO adsorption, as feldspar contains K^+^, Na^+^, or Ca^2+^ ions that are known to interact with the sulfonate moiety contained in MO and AR14, possibly leading to stronger adsorption. Strong adsorption of pollutants could be harmful for depollution by photocatalysis. With the increase of adsorbed MO on the surface of the nanostructures, a congregation effect could occur and inhibit water adsorption and OH^●^ formation. Therefore, as OH^●^ ions are the main oxidants for MO degradation in the photocatalytic process^29^, this could explain the change in the photocatalytic efficiency. However, in this case, AR is supposed to be better adsorbed than MO due to their respective sulfonate moieties. Considering that AR degradation is relatively easily carried out directly by photooxidation with the hole of the electron–hole pairs^30^, the congregation effect would be less important, and the efficiency of the AR degradation would not lessen.

It is nevertheless important to highlight the good activity of both civil engineering materials for any dye and the good reproducibility, as shown by the average gap in the first degradation efficiency of the ZnO nanostructures. For each dye, samples were reused several times with an annealing regeneration treatment at 350 °C for 30 min. The sample after annealing at 350 °C for 30 min when used for other tests can be considered a new photocatalyst surface (named cycle 1)^27^.

**Fig. 4:**
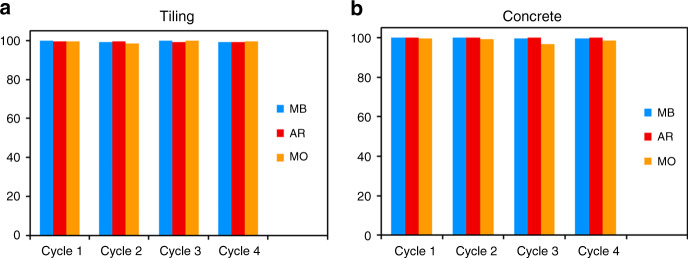
Durability of the ZnO NW photocatalyst. Degradation rate of the different dyes by our photocatalysts for subsequent experiment cycles after 3 h using ZnO-decorated **a** tiling and **b** concrete

To ensure the viability of ZnO-decorated civil engineering materials in daily life, we must ensure that they maintain their efficiency over time, even without any surface treatment, especially heating treatments, which could damage the material in the long run. Therefore, the stability of the samples was investigated over four photodegradation cycles without regeneration between each experiment, as described in the Experimental section. Figure 4a, b shows no loss in photocatalytic activity after four cycles of 3 h under UV illumination without intermediate regeneration for each sample and for any dye. After 8 months under storage conditions without special protection, the concrete covered by ZnO NWs was arbitrarily used for MB degradation to verify the viability of the ZnO-decorated civil engineering materials after a long time in the atmosphere. No losses of efficiency were observed (results not shown in this paper). This result implies that our samples could be used as sustainable civil engineering materials for environmental depollution.

## Conclusion

In this work, we successfully synthesized ZnO NWs onto commercially available civil engineering materials using a hydrothermal synthesis method. This easy and low-cost method allowed us to obtain an almost homogeneous repartition of our nanostructures on the entirety of the surface of our substrates, as shown by the SEM images. The measured gap values were similar to those of the ZnO NWs grown on typical substrates, i.e., ~3.18 eV and 3.20 eV for concrete and tiling, respectively. The excellent photocatalytic efficiency of our samples was demonstrated on three commonly used dyes (MO, MB, and AR 14). All of the dyes were fully degraded, in less than 2 h for MB and AR 14, and less than 3 h for the more difficult to degrade MO. Investigating the durability of our samples, we found very promising results, as they showed no loss of efficiency after four experiment cycles. The ability of implementing ZnO NWs on civil engineering materials, their good photocatalytic properties, and the possibility to re-use samples with minimal efficiency losses, even after several months, are very promising for the use of our nanostructures as road surfaces for air or water depollution.

## Materials and methods

### Substrate preparation and ZnO nanowire growth

Red concrete pavement and gray floor tiling, with initial commercial dimensions of 10 × 10 × 8 cm and 15 × 20 × 0.5 cm, respectively (purchased from DIY retailer Leroy Merlin, France), were used as the substrates and subsequently cut into pieces of ~2.5 × 2.5 × 0.5 cm for laboratory synthesis. Prior to any synthesis, each sample underwent a standard washing process to remove any dust and impurities adsorbed onto the surface, followed by annealing at 350 °C for 30 min to remove the remaining water in their porous structure.

Following this preparation, ZnO NWs were grown onto our civil engineering samples by a two-step hydrothermal method, slightly adapted from our previous works^13,17^. In contrast to the previously described protocols, the classical spin-coating process is not suitable for those substrates. Indeed, due to their large surface roughness, the coating deposition would not be perfectly homogeneous on such materials. To avoid this, our simple method was adapted from a previous impregnation method. First, our samples were impregnated with a buffer solution (C = 0.01 M) of Zn(Ac)_2_ (zinc acetate dihydrate, 99%, VWR) in absolute ethanol (99.9%, VWR). The impregnation (of brief duration) was repeated four times and followed by thermal annealing at 350 °C for 30 min. Then, the second step consisted of the hydrothermally driven growth of ZnO NWs in an autoclave containing an aqueous mixture of 0.05 M of methenamine (HMTA, ≥99%, VWR) and 0.05 M of Zn(NO_3_)_2_ (zinc nitrate hexahydrate, 98%, Sigma-Aldrich) at 90 °C for 4 h.

Our “horizontal dip” process (distinguished from the classical dip-coating process, which is usually performed in a vertical position), which is easy to reproduce and has a shorter calcination time than the usual PVA process^17^, is a suitable way to promote growth on the concrete and tiling surfaces without damage to the nonconventional substrates. It is worth mentioning that the short time (30 min) in the furnace should not affect the mechanical properties of our samples, as the properties of concrete are not modified by temperatures lower than 400 °C^31^.

After the ZnO NW growth, the concrete or tiling samples covered by ZnO nanostructures were washed with DI water, dried under a hot air flow, and then annealed in an oven at 350 °C for 30 min to remove any hydroxide residue and to improve the ZnO crystallinity. It is also worth noting that no damage appeared on our materials after the synthesis, as the hydrothermal synthesis was performed at low temperature (90 °C), and the post annealing step has a short duration (30 min).

The obtained samples were then characterized by UV–visible spectroscopy (Maya2000 Pro from Ocean Optics) and by field-emission scanning electron microscopy (Zeiss FE-SEM NEON 40) to determine their bandgap value and to investigate the morphology of the obtained ZnO nanostructures.

### Photocatalysis for water purification

To demonstrate the photocatalytic performance of these new materials, we carried out the photodegradation of model organic molecules commonly used in the pharmaceutical, food, and textile industries. The selected molecules were three organic dyes: acid red 14 (50%, Sigma-Aldrich), methylene blue (Certistain®, Sigma-Aldrich), and methyl orange (85%, Sigma-Aldrich). The corresponding aqueous solutions were prepared with concentrations fixed at 10 µM. Samples of ZnO-decorated civil engineering materials were immersed into 30 mL of dye-contaminated aqueous solution and irradiated with a UV lamp (Hamamatsu LC8, 4500 mW/cm^2^, *λ* = 365 nm) under magnetic stirring. The distance between the UV source and the sample surface was maintained at 10 cm.

The as-described photocatalysis was monitored by UV–visible spectrophotometry (PerkinElmer Lambda 35) and recorded every 15 min. The residual dye content was determined by the degradation efficiency (also called the degradation rate), defined as X:

1 $$X({\mathrm{\% }}) = \frac{{A_0 - A}}{{A_0}} \times 100$$

where *A*_*0*_ and *A* stand for the initial and actual absorption peak values, respectively, at the wavelength of the maximum absorption for each dye (i.e., *λ*_max_ = 665 nm for MB; 464 nm for MO; and 515 nm for AR14).

To verify the durability of both samples, we reused the same sample for multiple tests. The sample after annealing at 350 °C for 30 min, even when used for other tests, can be considered a new photocatalyst surface (named cycle 1). Five cycles were repeated for each understudied organic dye without any thermal treatment between two successive cycles. The sample was only washed using DI water and dried under hot air flow after each depollution process before being re-employed in the next depollution cycle under identical conditions.
